# The association between the atherogenic index of plasma and all-cause mortality in patients undergoing peritoneal dialysis: a multicenter cohort study

**DOI:** 10.1186/s12944-025-02510-z

**Published:** 2025-03-13

**Authors:** Yaohua Hu, Liming Yang, Zhanshan Sun, Xiaoxuan Zhang, Xueyan Zhu, Jian Li, Xinyang Li, Mengyuan Yu, Wenpeng Cui

**Affiliations:** 1 Department of Nephrology, The Second Hospital of Jilin University, No. 4026 Yatai Street, Changchun, 130041 Jilin Province China; 2 Department of Nephrology, The First Hospital of Jilin University-the Eastern Division, Changchun, 130041 Jilin Province China; 3 Department of Nephrology, Xing’anmeng People’s Hospital, Ulan Hot 137400, Inner Mongolia Autonomous Region, China; 4 Department of Nephrology, Jilin FAW General Hospital, 130041 Changchun, Jilin Province China; 5 Department of Nephrology, Jilin Central Hospital, 132011 Jilin, Jilin Province China

**Keywords:** Atherogenic index of plasma, Peritoneal dialysis, All-cause mortality

## Abstract

**Background:**

The Atherogenic Index of Plasma (AIP) has been reported as a strong predictor of all-cause mortality in the overall population. However, the lipid profile changes in individuals with end-stage kidney disease (ESKD) undergoing peritoneal dialysis (PD) may affect the prognostic utility of AIP for all-cause mortality. The connection between them remains unclear.

**Methods:**

This study included patients receiving PD at five hospitals in China from January 1, 2013, to December 31, 2019, with follow-up until June 30, 2020. The primary exposure variable in this investigation was the logarithm of the triglycerides (TG)/high-density lipoprotein cholesterol (HDL-C) ratio, which was used to compute the AIP, and the outcome variable was all-cause mortality. A Cox proportional hazards regression model was employed to analyze the association between AIP and all-cause mortality. Moreover, stratified analyses were performed to investigate this association further. Kaplan-Meier curves were employed for survival analysis, assessing the prognostic implications of varying AIP levels. Nonlinear associations were examined using smooth curve fitting techniques.

**Results:**

A total of 869 patients were included in this study, of whom 153 died during the follow-up period. An inverse association was observed between AIP and all-cause mortality risk in the highest tertile compared to the lowest tertile (HR: 0.56, 95% CI: 0.37–0.84) after correcting for potential confounding variables. Moreover, a nonlinear association was observed between the rates of all-cause mortality and AIP. A segmented Cox regression model identified an inflection point at an AIP value of 0.63 (*P* = 0.014 for the log-likelihood ratio test). More specifically, it was negatively associated with the all-cause mortality risk (HR: 0.42, 95% CI: 0.25–0.73, *P* = 0.002) when AIP was ≤ 0.63. On the other hand, AIP showed a positive association with the risk of all-cause mortality when it was more than 0.63 (HR: 8.94, 95% CI: 1.66–48.10, *P* = 0.011).

**Conclusion:**

The present study identified a non-linear association between AIP and all-cause mortality in patients receiving peritoneal dialysis.

**Supplementary Information:**

The online version contains supplementary material available at 10.1186/s12944-025-02510-z.

## Introduction

End-stage kidney disease (ESKD) is a critical global health issue, imposing a significant burden on healthcare systems [1, 2]. From 2000 to 2019, the prevalence rate of ESKD among Asians increased by 149.5% [3]. Hemodialysis (HD) and peritoneal dialysis (PD) are the two primary treatment modalities for ESKD [4, 5]. PD has gained widespread acceptance due to its hemodynamic stability, better preservation of residual renal function, and the convenience of home-based administration [6].

Dyslipidemia, characterized by elevated low-density lipoprotein cholesterol (LDL-C), reduced high-density lipoprotein cholesterol (HDL-C), and higher triglycerides (TG), is frequently seen in patients undergoing PD [7, 8]. Dyslipidemia is associated with poor survival outcomes, particularly in cases when low HDL-C values and elevated TG levels are present together [9, 10]. Furthermore, Gaziano et al. [11] first identified elevated TG levels combined with low HDL-C as a potent predictor of the development of unfavorable cardiovascular events.

The atherogenic index of plasma (AIP) is associated with atherosclerosis [12], cardiovascular events [10], insulin resistance, and glucose metabolism disorders [13]. Furthermore, it has been reported that in hypertensive people, a higher AIP is directly associated with the degree of arteriosclerosis [14]. Moreover, in the general population [15], as well as in diabetic [9] and elderly populations [16], AIP showed a significant association with mortality rates.

In a Korean cohort study by Mi Jung Lee et al. [17] including 740 HD patients and 434 PD patients, a nonlinear association was seen between the AIP and survival probability in dialysis patients. While lipid profiles differ between HD and PD patients, data on the impact of AIP on all-cause mortality in PD patients remain limited. The complex association between AIP and all-cause mortality in patients undergoing PD requires further investigation. As a result, this study employs a multicenter cohort design to evaluate the association between AIP and all-cause mortality in PD patients.

## Methods

### Study design and participants

**Fig. 1:**
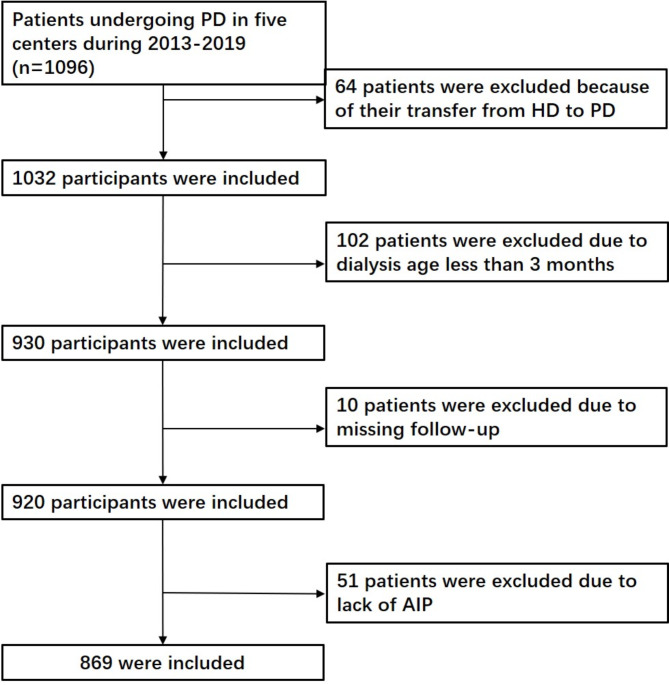
Flowchart of the study population. Abbreviations: AIP, atherogenic index of plasma; PD, peritoneal dialysis; HD, hemodialysis

Adult ESKD patients receiving PD between January 1, 2013, and December 31, 2019, were recruited from five medical facilities: the Xing’anmeng People’s Hospital in Inner Mongolia, the Second Department of the First Hospital of Jilin University, Jilin Central Hospital, Jilin First Automobile General Hospital, and the Second Hospital of Jilin University. Exclusion criteria included patients who transitioned from HD to PD, those with survival of less than three months, and those with incomplete data. All participants were monitored until death, kidney transplantation, or June 30, 2020, whichever occurred first. Patients received information about the available options for renal replacement therapy, with the final decision resting with them. A detailed flowchart illustrating patient recruitment is provided in Fig. 1. Ethical approval was obtained from the Ethics Committee of Jilin University’s Second Hospital (Approval No. 2020031). As this study was retrospective, informed consent was not required.

### Data collection

Demographic information, baseline laboratory indicators, and clinical outcomes of patients undergoing PD were collected. Demographic data included age, sex, primary causes of ESKD, and comorbidities such as diabetes and hypertension. Primary causes of ESKD include chronic glomerulonephritis, diabetic nephropathy, hypertension-related kidney disease, and other disorders. Baseline laboratory indicators collected within two days before PD initiation included hemoglobin, albumin, TG, LDL-C, HDL-C, total cholesterol (TC), uric acid, blood urea nitrogen (BUN), the estimated glomerular filtration rate (eGFR), and serum creatinine.

### Definitions of variables

The exposure variable, AIP, is defined as lg[TG (mmol/L)/HDL-C (mmol/L)], with TG and HDL-C measured in mmol/L. HDL-C levels were determined *via* precipitation or direct immunoassay. Fasting venous blood samples were collected from each participant to quantify TG. Patients were stratified into tertiles based on AIP values. All-cause mortality was the primary endpoint, with follow-up concluding on June 30, 2020, or upon events such as death, kidney transplantation, transition to HD, transfer to another healthcare facility, or loss to follow-up.

### Statistical analysis

Participants were stratified into tertiles based on AIP levels: T1 (< 0.0145), T2 (0.015–0.280), and T3 (> 0.280). Categorical variables such as gender, primary disease, diabetes, and hypertension were presented as frequencies and percentages, while continuous variables were expressed as means (± SD) for normally distributed data or medians (interquartile range) for non-normally distributed data. Statistical tests, including the Student’s *t*-test, Mann-Whitney *U* test, or χ² test, were used to evaluate differences among groups. Kaplan-Meier curves were used to evaluate survival rates across AIP tertiles. Cox proportional hazards models were employed to assess the association between AIP and all-cause mortality, with subgroup analyses based on gender, age, diabetes, study centers, and hypertension graphically represented in forest plots. Smooth curve fitting and piecewise Cox regression models were used to explore nonlinear associations and the threshold influence of AIP on the probability of all-cause mortality. Data analysis was conducted using R (version 4.2.0) and Empower Stats (version 4.2), with GraphPad Prism 9.0 for plotting. A *P*-value < 0.05 was considered statistically significant.

## Results

### Baseline characteristics

Of the 1,096 PD patients screened from five hospitals between 2013 and 2019, 869 were included in the study. A total of 227 patients were excluded due to transitioning from HD to PD, incomplete data, loss of follow-up, or a survival period of less than three months (Fig. 1). The patients were divided into tertiles based on their AIP levels. Among the included participants, 60.07% were male, with an average age of 54 years (43–63). The primary causes of ESKD included chronic glomerulonephritis (36.82%), diabetic nephropathy (31.76%), and hypertensive nephropathy (15.77%).

Patients with higher AIP levels showed lower HDL-C levels and higher TG, TC, and LDL-C levels, as well as a greater prevalence of diabetes (*P* < 0.05). No significant differences were observed across groups for age, gender, hemoglobin, albumin, BUN, serum creatinine, or hypertension (*P* > 0.05; Table 1).

**Table 1:** Baseline characteristics of subjects categorized by the atherogenic index of plasma

Variables	Total	T1 < 0.0145	T2 0.015–0.280	T3 > 0.280	*P*-value
No. of participants	869	290	289	290	
Age (year)	54.00 (43.00–63.00)	55.50 (44.00–65.00)	52.00 (40.00–63.00)	54.00 (44.00–61.00)	0.052
Gender, *n*(%)					0.151
Male	522 (60.07%)	177 (61.03%)	161 (55.71%)	184 (63.45%)	
Female	347 (39.93%)	113 (38.97%)	128 (44.29%)	106 (36.55%)	
Primary renal diseases, *n*(%)					0.007
Glomerulonephritis	320 (36.82%)	115 (39.66%)	117 (40.48%)	88 (30.34%)	
Diabetic nephropathy	276 (31.76%)	76 (26.21%)	84 (29.07%)	116 (40.00%)	
hypertensive renal disease	137 (15.77%)	55 (18.97%)	43 (14.88%)	39 (13.45%)	
Others	136 (15.65%)	44 (15.17%)	45 (15.57%)	47 (16.21%)	
Hemoglobin (g/l)	84.00 (73.00–98.00)	84.00 (72.00–99.00)	83.00 (72.00–95.00)	85.50 (74.00-100.00)	0.071
Albumin (g/l)	34.26 ± 5.95	34.08 ± 5.93	34.38 ± 6.33	34.33 ± 5.59	0.816
TG (mmol/L)	1.42 (0.97–1.99)	0.82 (0.68–1.05)	1.40 (1.20–1.61)	2.29 (1.88–3.04)	< 0.001
TC (mmol/L)	4.31 (3.55–5.11)	4.03 (3.43–4.81)	4.34 (3.67–5.13)	4.49 (3.69–5.30)	< 0.001
HDL-C (mmol/L)	1.00 (0.82–1.24)	1.27 (1.07–1.50)	1.00 (0.84–1.16)	0.81 (0.68–0.96)	< 0.001
LDL-C (mmol/L)	2.60 (2.04–3.27)	2.42 (1.95–2.89)	2.68 (2.19–3.35)	2.75 (2.04–3.47)	< 0.001
BUN (mmol/L)	24.70 (17.80–32.90)	24.70 (17.83–33.59)	25.66 (19.40–33.20)	23.88 (16.60–31.70)	0.084
Uric acid (umol/L)	441.00 (376.00-519.00)	440.50 (368.10-500.75)	442.00 (385.00-546.00)	441.00 (373.68–519.00)	0.022
Serum creatinine (umol/L)	776.75 (618.00-992.00)	759.20 (586.49-988.15)	808.77 (636.80–1042.00)	769.40 (617.55-965.15)	0.166
eGFR (ml/min/1.73 m2)	5.39 (4.10–7.36)	5.43 (3.96–7.87)	5.16 (4.11–6.85)	5.69 (4.29–7.37)	0.107
Hypertension, *n*(%)					0.982
Yes	799 (91.94%)	267 (92.07%)	265 (91.70%)	267 (92.07%)	
no	70 (8.06%)	23 (7.93%)	24 (8.30%)	23 (7.93%)	
Diabetes, *n*(%)					< 0.001
Yes	330 (37.97%)	93 (32.07%)	100 (34.60%)	137 (47.24%)	
no	539 (62.03%)	197 (67.93%)	189 (65.40%)	153 (52.76%)	
Mechanical complications, *n*(%)					0.509
Yes	93 (10.70%)	29 (10.00%)	28 (9.69%)	36 (12.41%)	
no	776 (89.30%)	261 (90.00%)	261 (90.31%)	254 (87.59%)	
Complications of infection, *n*(%)					0.867
Exit-site infection	6 (2.26%)	3 (3.26%)	2 (2.33%)	1 (1.15%)	
Tunnel infection	5 (1.89%)	2 (2.17%)	1 (1.16%)	2 (2.30%)	
Peritonitis	254 (95.85%)	87 (94.57%)	83 (96.51%)	84 (96.55%)	
Method of catheter placement, *n*(%)					0.526
Surgical incision method	166 (57.24%)	166 (57.24%)	164 (56.75%)	170 (58.62%)	
Percutaneous puncture method	121 (41.72%)	121 (41.72%)	120 (41.52%)	113 (38.97%)	
Laparoscopic method	2 (0.69%)	2 (0.69%)	5 (1.73%)	7 (2.41%)	
missing	1 (0.34%)	1 (0.34%)	0 (0.00%)	0 (0.00%)	

Abbreviation: TG triglyceride, TC total cholesterol, LDL-C low-density lipoprotein cholesterol, HDL-C high-density lipoprotein cholesterol, BUN blood urea nitrogen, eGFR, estimated glomerular filtration rate.

### Analysis of Kaplan-Meier survival curves

**Fig. 2:**
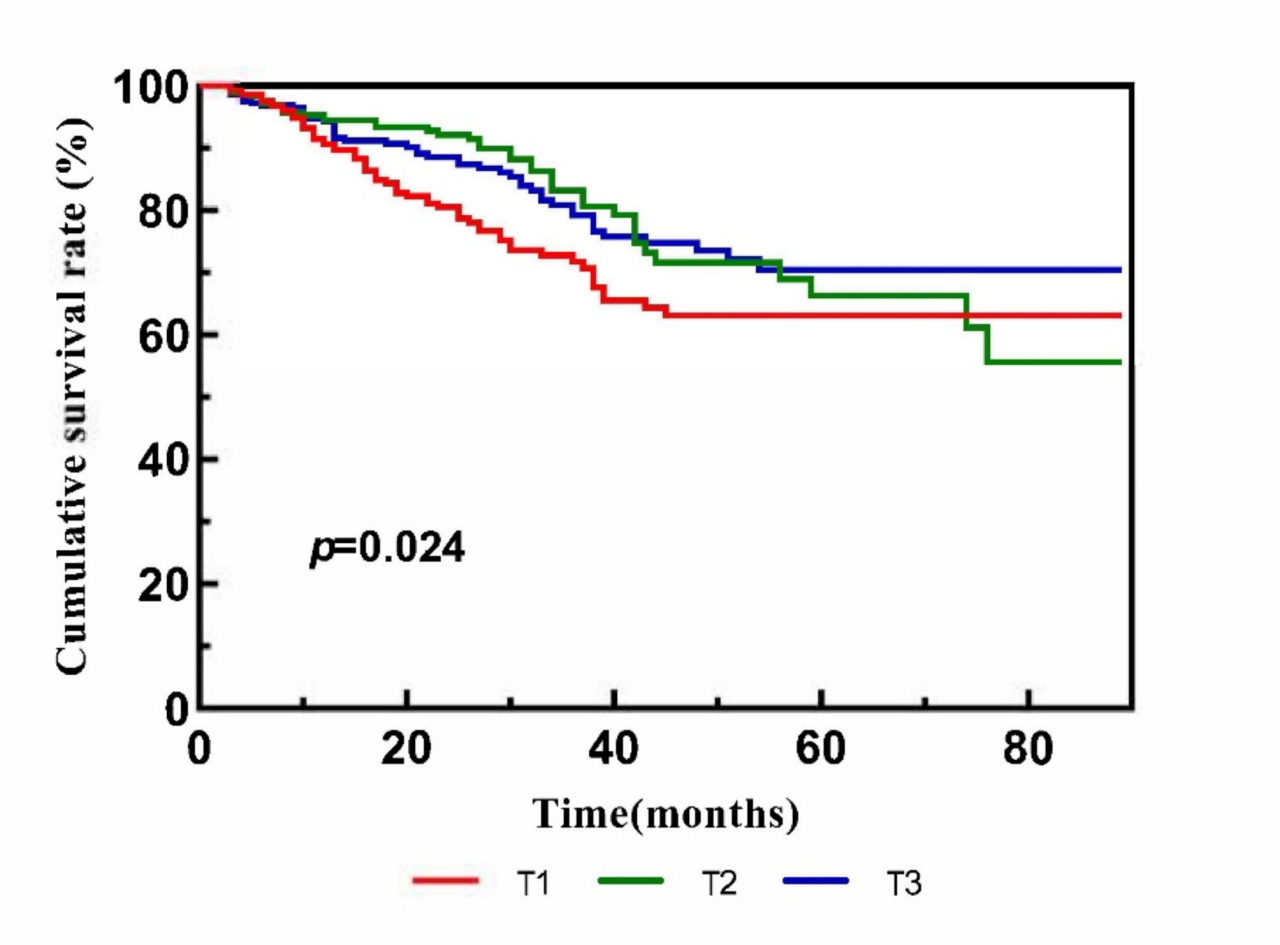
K-M curves for all-cause mortality delineated by the tertiles of the AIP

During the follow-up period, 153 deaths were reported. The specific causes of death are listed in Table S3. The Kaplan-Meier analysis revealed a significant reduction in all-cause mortality in patients with higher AIP levels (T3) compared to the lowest tertile (T1) (log-rank test, *P* = 0.024; Fig. 2).

### Association between the AIP and overall mortality

The multicollinearity assessment (Table S1) indicated an absence of any significant multicollinearity among the covariables, with the variance inflation factors (VIFs) for the covariables all being < 5. The association between AIP and all-cause mortality is presented in Table 2. In Model 1 (unadjusted), elevated AIP levels were associated with a reduced risk of all-cause mortality. Model 2 adjusted for gender, age, while Model 3 included further covariables such as diabetes, uric acid, serum creatinine, BUN, hemoglobin, albumin, method of catheter placement, and eGFR.

After complete adjustments in Model 3, patients with AIP > 0.28 showed a 44% lower risk of all-cause mortality (HR, 0.56 [95% CI, 0.37–0.84]) compared to those with AIP < 0.0145.

**Table 2:** The association between AIP and the risk of all-cause mortality in patients undergoing peritoneal dialysis

Exposure	No. of patients	Model 1 HR (95%CI)	Model 2 HR (95%CI)	Model 3 HR (95%CI)
AIP (Tertile)				
T1	290	1.00 (ref)	1.00 (ref)	1.00 (ref)
T2	289	0.63 (0.42, 0.93)	0.70 (0.47, 1.04)	0.59 (0.39, 0.90)
T3	290	0.65 (0.45, 0.95)	0.66 (0.45, 0.96)	0.56 (0.37, 0.84)
P for trend		0.0237	0.0295	0.0050

Model 1: Unadjusted

Model 2: adjusted for gender, age

Model 3: adjusted for model 2 covariables and Diabetes, Hemoglobin, Blood urea nitrogen, Serum creatinine, Albumin, Uric acid, Method of catheter placement, and eGFR

Abbreviations: AIP, atherogenic index of plasma; HR, hazard ratio; CI, confidence interval;

### Subgroup analyses

**Fig. 3:**
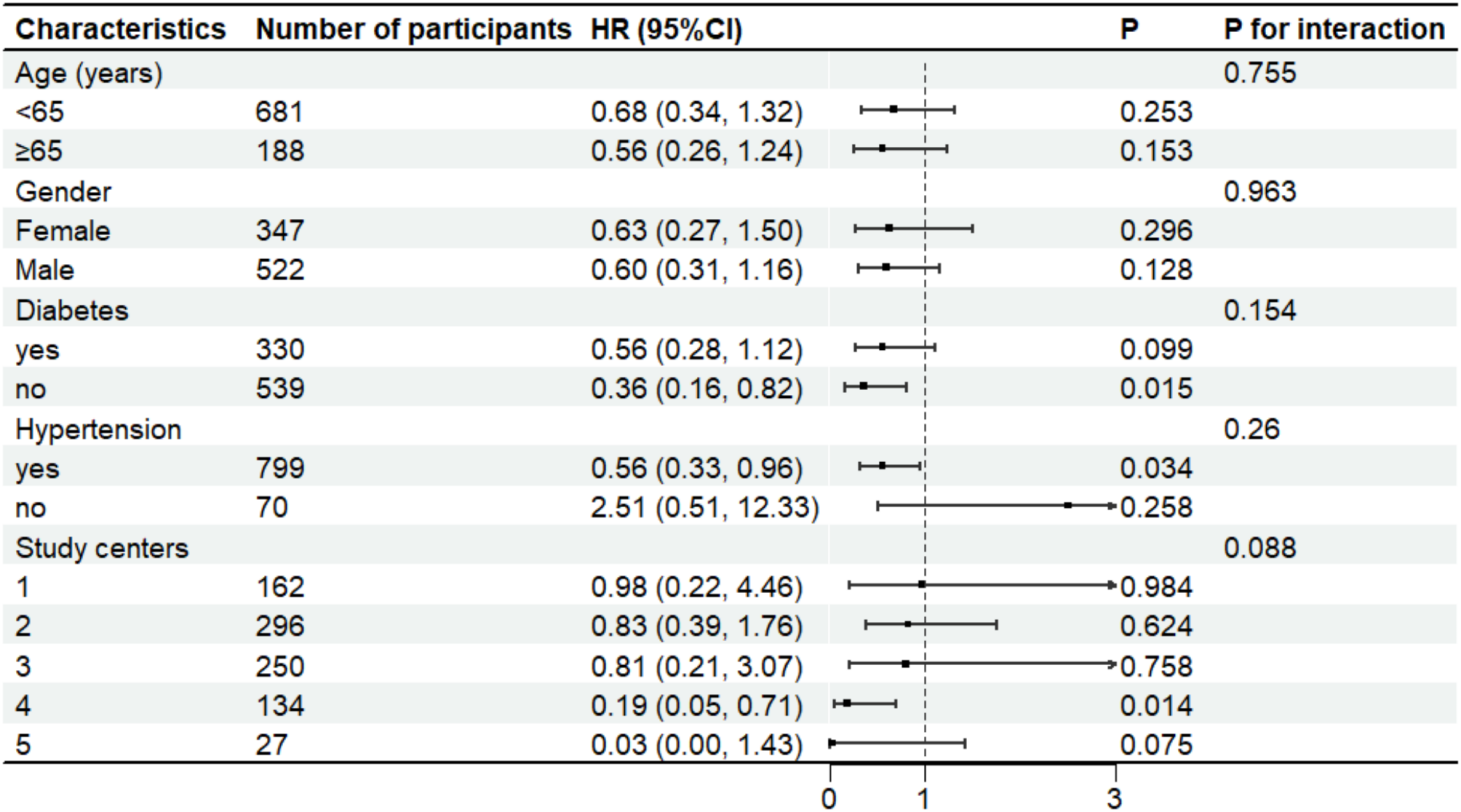
Forest plot illustrating the association between the AIP and all causes mortality across several categories. Abbreviations: HR, hazard ratios; CI, confidence interval;

Subgroup analyses were conducted based on sex, age (≤ 65 years), diabetes, hypertension status, and study centers. The association between AIP and all-cause mortality was consistent across subgroups. Variables such as sex, age, diabetes, hypertension, and study centers showed no interactions (p for interaction > 0.05)( Fig. 3).

### Smoothing graphs and threshold effect analyses

**Fig. 4:**
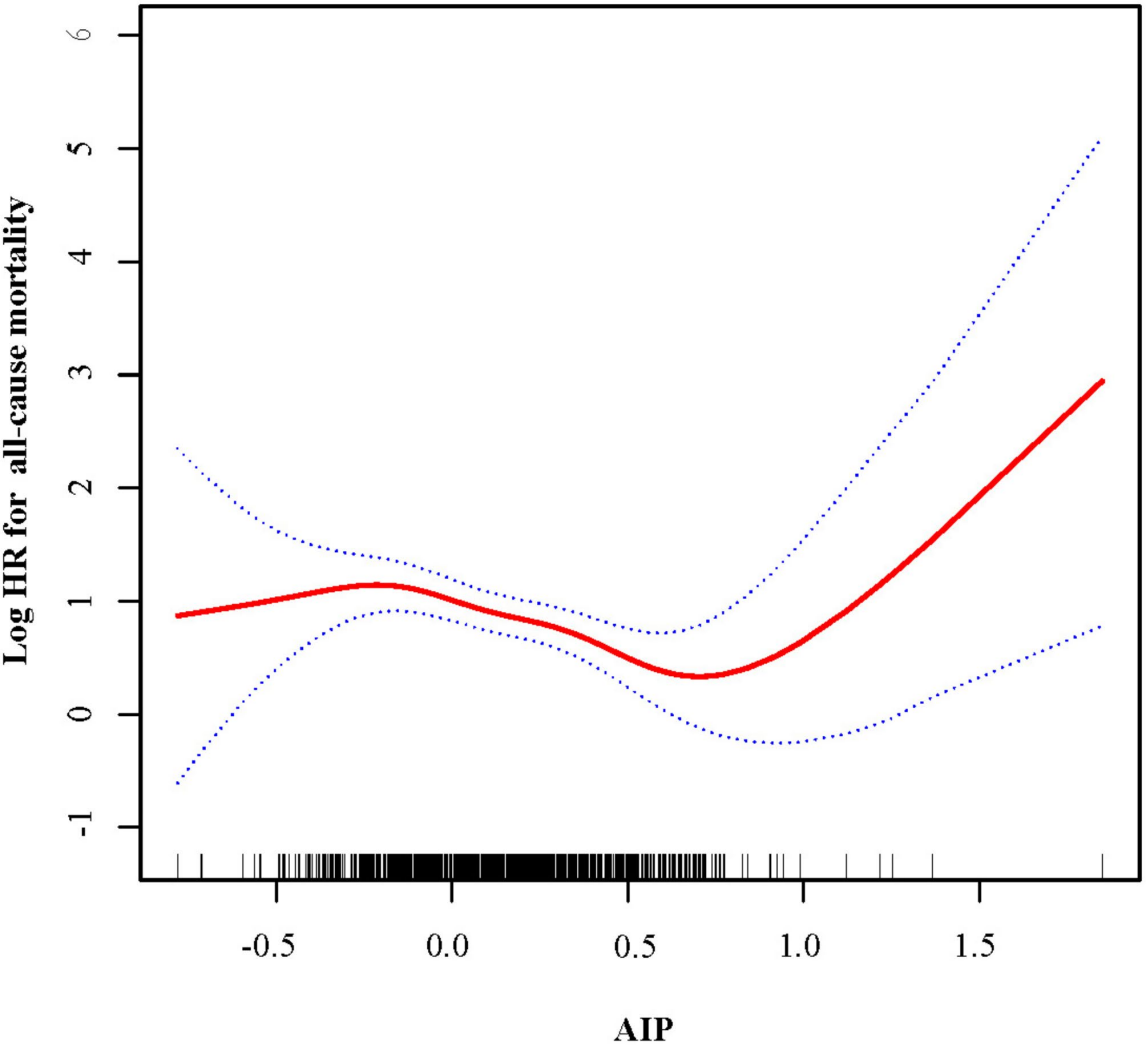
The nonlinear association between the AIP and the risk of all causes mortality in patients undergoing peritoneal dialysis. A nonlinear association was identified after controlling for gender, age, hypertension, and diabetes

Smooth curve fitting adjusted for covariables, including gender, age, diabetes, and hypertension, revealed a significant nonlinear association between AIP and all-cause mortality (*P* for non-linearity = 0.014; Fig. 4).

Threshold effect analysis identified an inflection point at an AIP value of 0.63 (Table 3). Below this threshold, each 1 mmol/L increase in AIP was associated with a 58% reduction in all-cause mortality risk (HR, 0.42 [95% CI, 0.25–0.73]). However, above the inflection point, each 1 mmol/L increase in AIP was associated with an 8.94-fold increase in mortality risk (HR, 8.94 [95% CI, 1.66–48.10]).

**Table 3:** Analysis of threshold effect

	HR (95%CI)	*p*
Inflection point	0.63	
≤ 0.63	0.42 (0.25, 0.73)	0.002
> 0.63	8.94 (1.66, 48.10)	0.011
P for log likelihood ratio test		0.014

We adjusted for gender, age, hypertension, and diabetes

Abbreviations: HR, hazard ratio; CI, confidence interval; AIP, atherogenic index of plasma;

## Discussion

To the best of our knowledge, this research is the first to establish an association between the AIP and all-cause mortality in patients undergoing PD. A higher AIP was significantly associated with a reduced risk of all-cause mortality, even after adjusting for potential confounding factors. This relationship remained robust across subgroups and demonstrated a nonlinear association, with an inflection point identified at an AIP value of 0.63. Below this threshold, higher AIP levels were associated with a reduced mortality risk, whereas above it, the risk of mortality increased.

AIP reflects the metabolic status of lipids and carbohydrates and has been widely investigated in various contexts [13]. Our findings align with data from previous research. Studies on HD patients reported an association between elevated AIP and improved survival [18, 19]. Similarly, studies conducted in Turkey found significant associations between AIP and non-obstructive coronary artery disease and adverse outcomes post-intervention for myocardial infarction [20, 21]. The U-shaped relationship identified in this study corroborates the findings from a South Korean cohort that enrolled 1,174 participants, which observed similar patterns in dialysis patients [17]. Moreover, Gulinuer Duiyimuhan’s study also indicated that both elevated and reduced levels of AIP were associated with elevated mortality rates among patients with hypertension. This study also observed a U-shaped association, particularly regarding cardiovascular mortality [22]. However, discrepancies exist in the literature, which may be attributed to differences in study population, methodology, and follow-up duration. For example, Minghui Qin et al. reported a J-shaped relationship with no statistically significant association between AIP and all-cause mortality beyond a specific threshold [23]. This discrepancy could be due to variations in the baseline characteristics of the study populations or differences in the statistical approaches used to model the association between AIP and mortality. Furthermore, a Chinese retrospective study on elderly PD patients associated elevated TG/HDL-C ratios, a surrogate for AIP, with higher mortality risk [24]. This finding contrasts with our results; a possible reason is that the study focused on a specific subgroup of elderly patients who were undergoing peritoneal dialysis and thus may have distinct metabolic profiles and risk factors compared to our cohort.

The underlying mechanisms explaining the association between AIP and mortality in PD patients remain uncertain, but there are a few possible explanations. The present study observed a nonlinear association between AIP and all-cause mortality, with a significant protective effect of higher AIP levels below the inflection point of 0.63. However, beyond this threshold, higher AIP levels were linked with an increased risk of death. This nonlinear association could explain these seemingly contradictory findings. Furthermore, malnutrition is relatively common in patients receiving dialysis [25], and higher lipid levels may indicate better nutritional status and energy reserves [26]. In addition, the phenomenon of “reverse epidemiology” in dialysis patients suggests that higher lipid levels may be associated with improved survival outcomes, contrary to what would be expected in the general population [27, 28, 29]. In addition, higher blood lipid levels may enhance the ability of patients to resist infection by supporting immune cell proliferation and functions, thereby reducing the incidence of peritoneal dialysis-related infectious complications and leading to better survival outcomes [30].

Our findings reveal that increasing AIP levels benefit all-cause mortality in PD patients when AIP is < 0.63, whereas elevated AIP levels above 0.63 correlate with poorer outcomes. However, it is important to note that the sample size for AIP > 0.63 is relatively small, and this finding was not validated in PD populations outside the five study centers included in this research. Therefore, while regular monitoring of AIP levels in PD patients is essential, particularly for those approaching or exceeding 0.63, the clinical significance of this cut-off should be interpreted with caution until further validation. Clinicians should encourage dietary modifications aimed at reducing saturated and trans fat intake while promoting regular physical activity. Future large-scale prospective cohort studies are required to validate these findings.

This study has some important strengths. First, it identifies a precise AIP inflection point of 0.63, offering novel insights into the association between AIP and all-cause mortality in PD patients. This threshold provides clinicians with a quantitative target for risk stratification. Second, these findings support the clinical utility of AIP as a practical biomarker to guide interventions such as dietary modifications and optimized treatment strategies for high-risk individuals. Lastly, the study addresses existing gaps in knowledge regarding AIP’s clinical application and thresholds, contributing to its effective use in managing dialysis patients.

Despite its strengths, this study has limitations. As a retrospective analysis, it is subject to information bias regarding laboratory indicators and precise time of death. The relatively small sample size, particularly for AIP > 0.63, may have limited the adjustment for all potential confounders, leaving residual confounding effects unaddressed. Moreover, the study population, confined to northern China, restricts the generalizability of the findings to other regions or ethnic groups. Our study utilized baseline levels of TG and HDL-C to calculate the AIP. The analysis based on the baseline data thus could not adequately account for the potential impact of dynamic changes in lipid profiles on mortality. This study did not consider potential confounding factors such as glucose load, ultrafiltration volume, peritoneal transport status, C-reactive protein levels, and medication use (such as statins and antihypertensives); future research should include these variables. These limitations highlight areas for future research, including larger, multi-regional cohort studies and the exploration of unexamined confounders.

## Conclusion

This study demonstrated a significant nonlinear association between AIP levels and all-cause mortality in PD patients, with an inflection point of 0.63. While AIP shows promise as a biomarker for risk stratification, there are as yet no interventional strategies based on AIP. Further research is needed to evaluate its clinical utility and potential role in guiding patient management.

## Electronic supplementary material

Below is the link to the electronic supplementary material.

Supplementary Material 1

Supplementary Material 2

Supplementary Material 3

## Data Availability

No datasets were generated or analysed during the current study.

## Declarations

### Abbreviations

AIP: Atherogenic Index of Plasma;

BUN: Blood urea nitrogen;

CI: Confidence interval;

eGFR: Estimated glomerular filtration rate;

ESKD: End-stage kidney disease;

HD: Hemodialysis;

HDL-C: High-density lipoprotein cholesterol;

HR: Hazard ratio;

LDL-C: Low-density lipoprotein cholesterol;

PD: Peritoneal dialysis;

TG: Triglycerides;

TC: Total cholesterol;
