# Supplementary figures and images for: The association between the atherogenic index of plasma and all-cause mortality in patients undergoing peritoneal dialysis: a multicenter cohort study
Source: Lipids Health Dis. 2025 Mar 13;24:91. doi: 10.1186/s12944-025-02510-z (PMC11905527; doi:10.1186/s12944-025-02510-z)

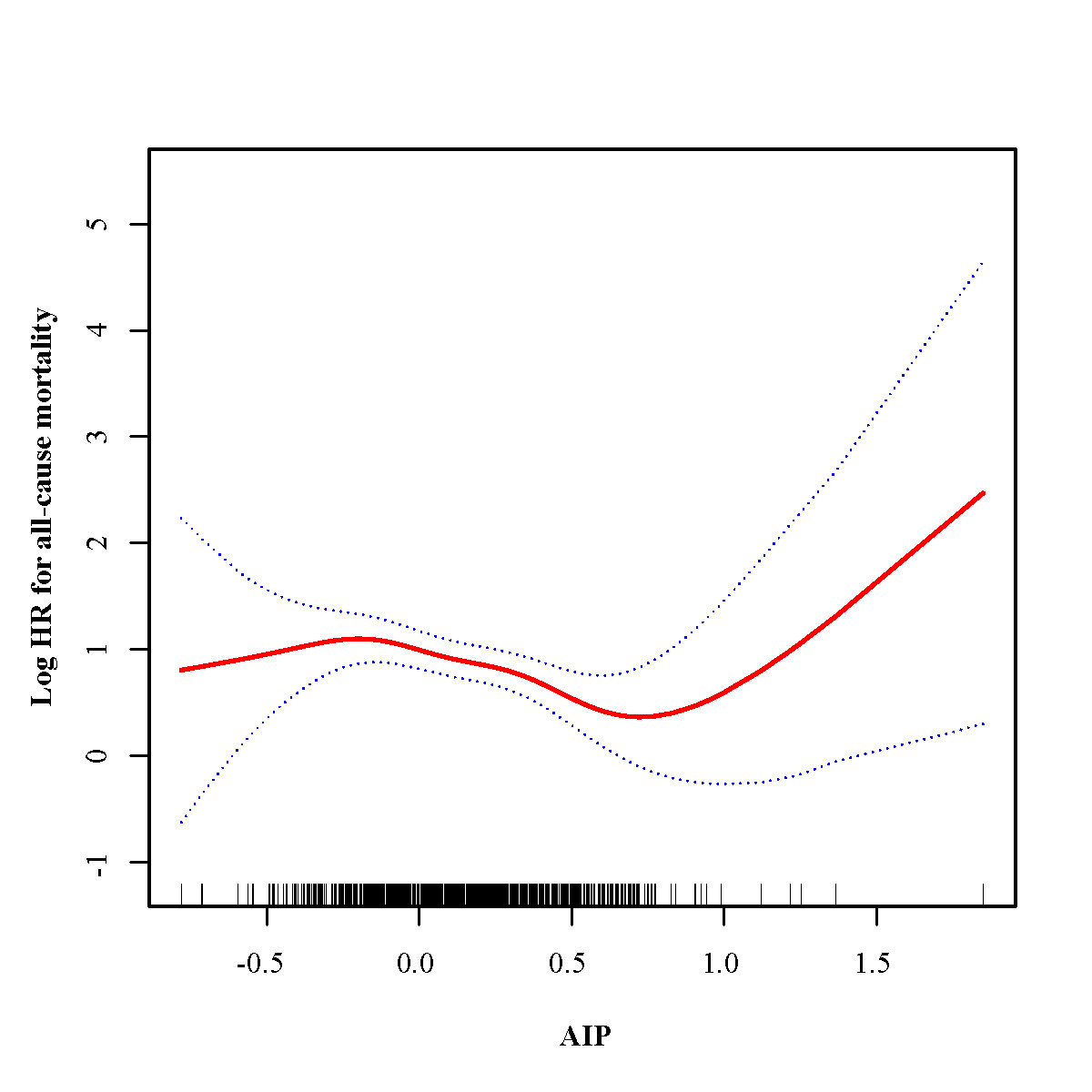

Supplement: Supplementary file 1 — Supplementary Material 1 [file 12944_2025_2510_MOESM1_ESM.jpg]

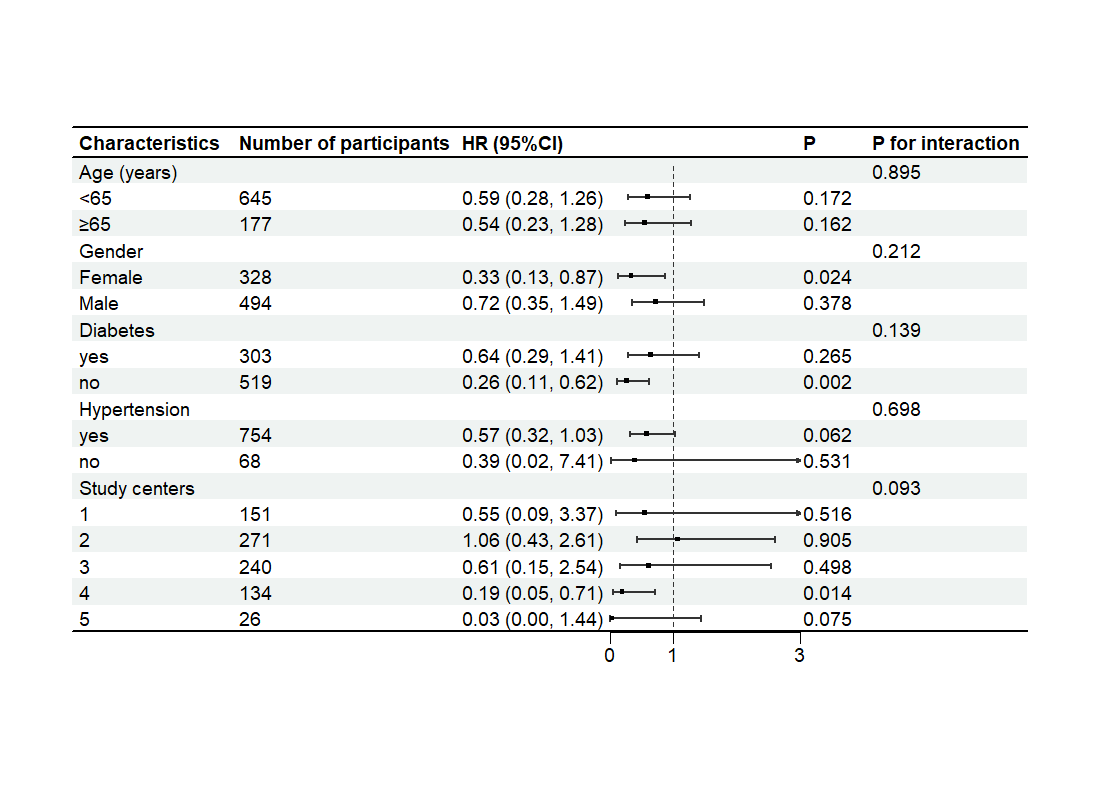

Supplement: Supplementary file 2 — Supplementary Material 2 [file 12944_2025_2510_MOESM2_ESM.tiff]
