# Supplementary material for: The association between the atherogenic index of plasma and all-cause mortality in patients undergoing peritoneal dialysis: a multicenter cohort study
Source: Lipids Health Dis. 2025 Mar 13;24:91. doi: 10.1186/s12944-025-02510-z (PMC11905527; doi:10.1186/s12944-025-02510-z)
Supplement: Supplementary file 3 — Supplementary Material 3 [file 12944_2025_2510_MOESM3_ESM.docx]

**TableS1.Collinearity Statistics.**

| **Variables** | **VIF** |
| --- | --- |
| AIP | 1.1 |
| Age | 1.2 |
| Gender | 1.2 |
| Hemoglobin | 1.3 |
| Albumin | 1.2 |
| Diabetes | 1.2 |
| Uric acid | 1.2 |
| Blood urea nitrogen | 1.4 |
| eGFR | 1.9 |
| Serum creatinine | 2.4 |
| TC | 1.4 |
| LDL-C | 1.3 |
| Hypertension | 1.0 |

**Table S2** **Analysis of threshold effect**

|  | HR (95%CI) | p |
| --- | --- | --- |
| Inflection point | 0.63 |  |
| ≤0.63 | 0.47 (0.27, 0.82) | 0.008 |
| >0.63 | 5.83 (1.06, 32.03) | 0.042 |
| P for log likelihood ratio test |  | 0.037 |

We adjusted for gender, age, hypertension, diabetes, hemoglobin, albumin, and eGFR.

**Table S3 Causes of Death Among Deceased Patients**

| **Cause of Death** | **Number of Patients (n)** | **Percentage (%)** |
| --- | --- | --- |
| Infections | 8 | 5.23% |
| Diseases of heart | 21 | 13.73% |
| Cerebrovascular diseases | 15 | 9.80% |
| Respiratory diseases | 4 | 2.61% |
| Digestive system diseases | 5 | 3.27% |
| Malignant neoplasms | 4 | 2.61% |
| All other causes | 96 | 62.75% |
| Total | 153 | 100% |

**Table S4 Classification of Mechanical Complications**

| **Classification** | **Number of Patients (n)** | **Percentage (%)** |
| --- | --- | --- |
| Peritoneal dialysate leakage | 7 | 7.53% |
| hernia | 6 | 6.45% |
| Intestinal perforation | 1 | 1.08% |
| Bleeding | 13 | 13.98% |
| Displacement of catheter | 61 | 65.59% |
| Greater omentum wrapping | 2 | 2.15% |
| Difficulty in getting in and out | 3 | 3.23% |
| Total | 93 | 100% |

**Table S5 The association of covariables with all-cause mortality was examined one by one**

| covariables | N | term | beta | Se. | exp(beta) | 95%CI Low | 95%CI Up | P-value |
| --- | --- | --- | --- | --- | --- | --- | --- | --- |
| Hemoglobin | 861 | Hemoglobin | 0.0100 | 0.0045 | 1.0101 | 1.0012 | 1.0190 | 0.0260 |
| Albumin | 858 | Albumin | -0.0323 | 0.0143 | 0.9682 | 0.9413 | 0.9958 | 0.0242 |
| Hypertension | 869 | Factor (Hypertension) | 0.2656 | 0.3640 | 1.3042 | 0.6390 | 2.6619 | 0.4656 |
| Diabetes | 869 | Factor (Diabetes) | 0.4138 | 0.1693 | 1.5126 | 1.0854 | 2.1079 | 0.0145 |
| Uric acid | 783 | Uric acid | -0.0035 | 0.0008 | 0.9965 | 0.9950 | 0.9980 | <0.0001 |
| BUN | 863 | BUN | -0.0578 | 0.0085 | 0.9438 | 0.9282 | 0.9597 | <0.0001 |
| Serum creatinine | 864 | Serum creatinine | -0.0020 | 0.0004 | 0.9980 | 0.9973 | 0.9987 | <0.0001 |
| eGFR | 862 | eGFR | 0.0640 | 0.0100 | 1.0661 | 1.0453 | 1.0873 | <0.0001 |
| LDL | 854 | LDL | -0.0395 | 0.0595 | 0.9613 | 0.8555 | 1.0801 | 0.5066 |
| TC | 861 | TC | 0.0436 | 0.0598 | 1.0445 | 0.9290 | 1.1744 | 0.4661 |
| Method of catheter placement | 868 | Factor (Percutaneous puncture method) | -1.2204 | 0.2014 | 0.2951 | 0.1989 | 0.4380 | <0.0001 |
|  |  | Factor (Laparoscopic method) | 0.0795 | 1.0104 | 1.0827 | 0.1494 | 7.8450 | 0.9373 |
| Mechanical complications | 869 | Factor (Mechanical complications) | 0.0334 | 0.2576 | 1.0340 | 0.6241 | 1.7132 | 0.8967 |
| Complications of infection | 869 | Factor (Exit-site infection) | 1.0479 | 0.7176 | 2.8517 | 0.6987 | 11.6394 | 0.1442 |
|  |  | Factor (Tunnel infection) | -0.6097 | 1.0090 | 0.5435 | 0.0752 | 3.9271 | 0.5457 |
|  |  | Factor (Peritonitis) | -0.0266 | 0.1727 | 0.9738 | 0.6942 | 1.3659 | 0.8776 |

**Table S6 The changes of the regression coefficients of AIP were observed by introducing the covariables in the basic model and removing the covariables in the full model**

|  | Basic model | Complete model |  |  |
| --- | --- | --- | --- | --- |
| covariables | +/- term | AIP | AIP | selection |
|  | Initial regression coefficient | -0.6350 | -0.6326 |  |
| Hemoglobin | Hemoglobin | -0.6844 | -0.6382 |  |
| Albumin | Albumin | -0.6283 | -0.6769 |  |
| Hypertension | factor(Hypertension) | -0.6406 | -0.6083 |  |
| Diabetes | factor(Diabetes) | -0.7377 * | -0.4841 * | Yes |
| Uric acid | Uric acid | -0.5405 * | -0.6927 | Yes |
| BUN | BUN | -0.7420 * | -0.5590 * | Yes |
| Serum creatinine | Serum creatinine | -0.5708 * | -0.6493 | Yes |
| eGFR | eGFR | -0.5480 * | -0.6434 | Yes |
| LDL | LDL | -0.6233 | -0.5845 |  |
| TC | TC | -0.6615 | -0.6034 |  |
| Method of catheter placement | factor(Method of catheter placement) | -0.5247 * | -0.7628 * | Yes |
| Mechanical complications | factor(Mechanical complications) | -0.6445 | -0.6340 |  |
| Complications of infection | factor(Complications of infection) | -0.6262 | -0.6365 |  |

* Represents a change of more than 10% from the starting regression coefficient

**Table S7 Schoenfeld residuals testing**

| **Variable** | **Chi-sq** | **df** | **p-value** |
| --- | --- | --- | --- |
| AIP | 0.043 | 1 | 0.837 |
| Gender | 0.653 | 1 | 0.419 |
| Age | 0.299 | 1 | 0.584 |
| Hemoglobin | 1.088 | 1 | 0.297 |
| Albumin | 0.15 | 1 | 0.698 |
| Hypertension | 1.057 | 1 | 0.304 |
| Diabetes | 0.003 | 1 | 0.959 |
| TC | 2.11 | 1 | 0.146 |
| LDL | 1.746 | 1 | 0.186 |
| Uric acid | 0.155 | 1 | 0.694 |
| Serum creatinine | 0.078 | 1 | 0.78 |
| Blood urea nitrogen | 0.74 | 1 | 0.39 |
| eGFR | 0.839 | 1 | 0.36 |
| Global | 11.053 | 13 | 0.606 |


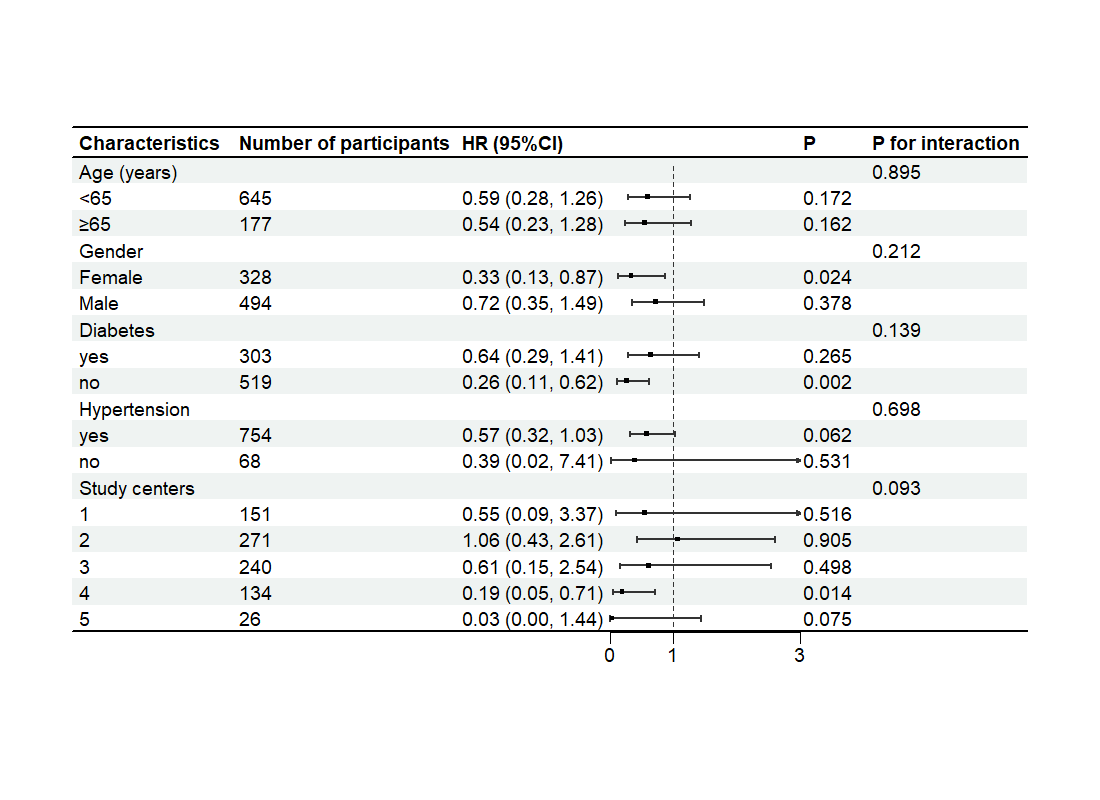


**Fig. S1** Forest plot illustrating the association between the AIP (<0.63) and all causes mortality across several categories.


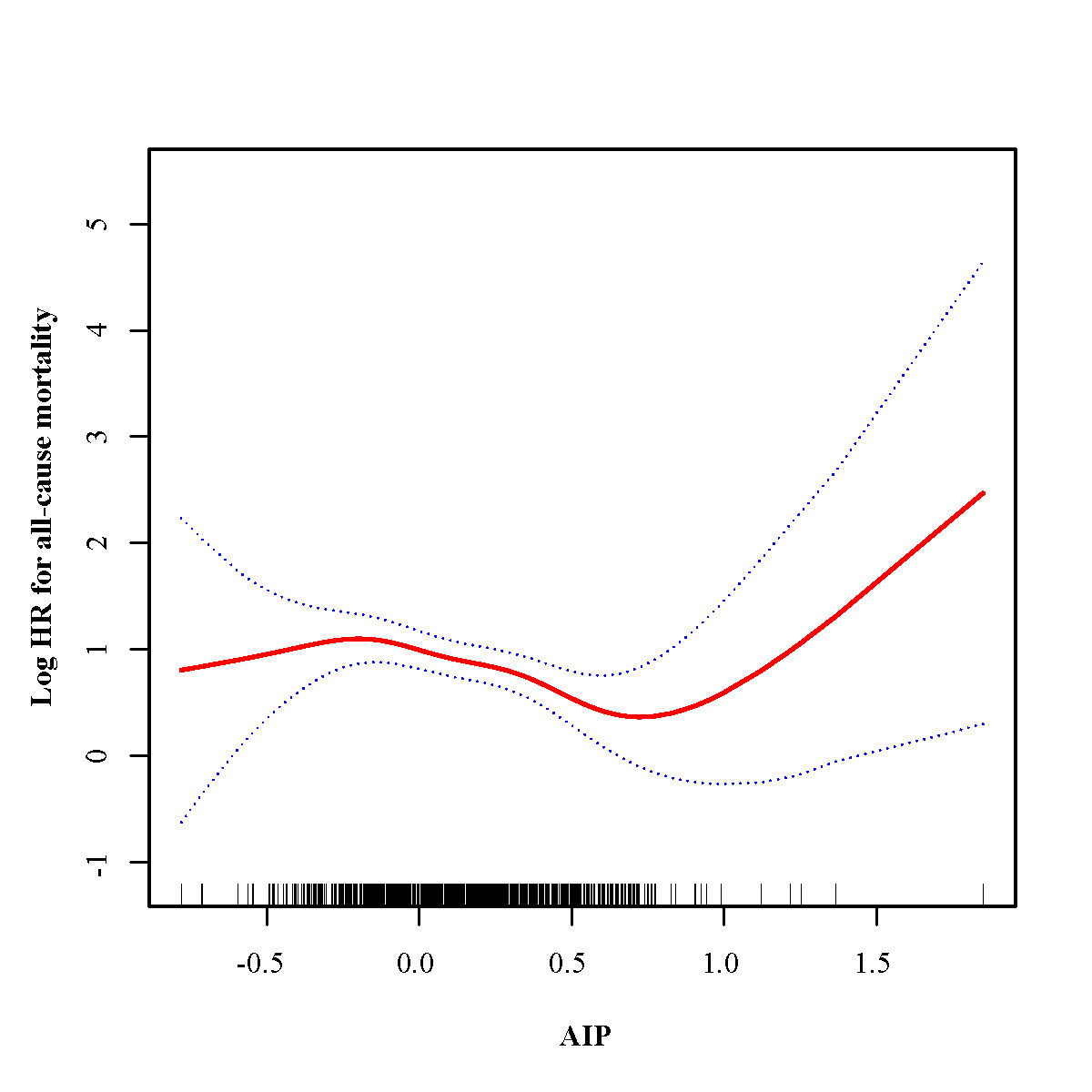


**Fig. S2** The nonlinear association between the AIP and the risk of all causes mortality in patients undergoing peritoneal dialysis. A nonlinear association was identified after controlling for gender, age, hypertension, diabetes, hemoglobin, albumin, and eGFR.


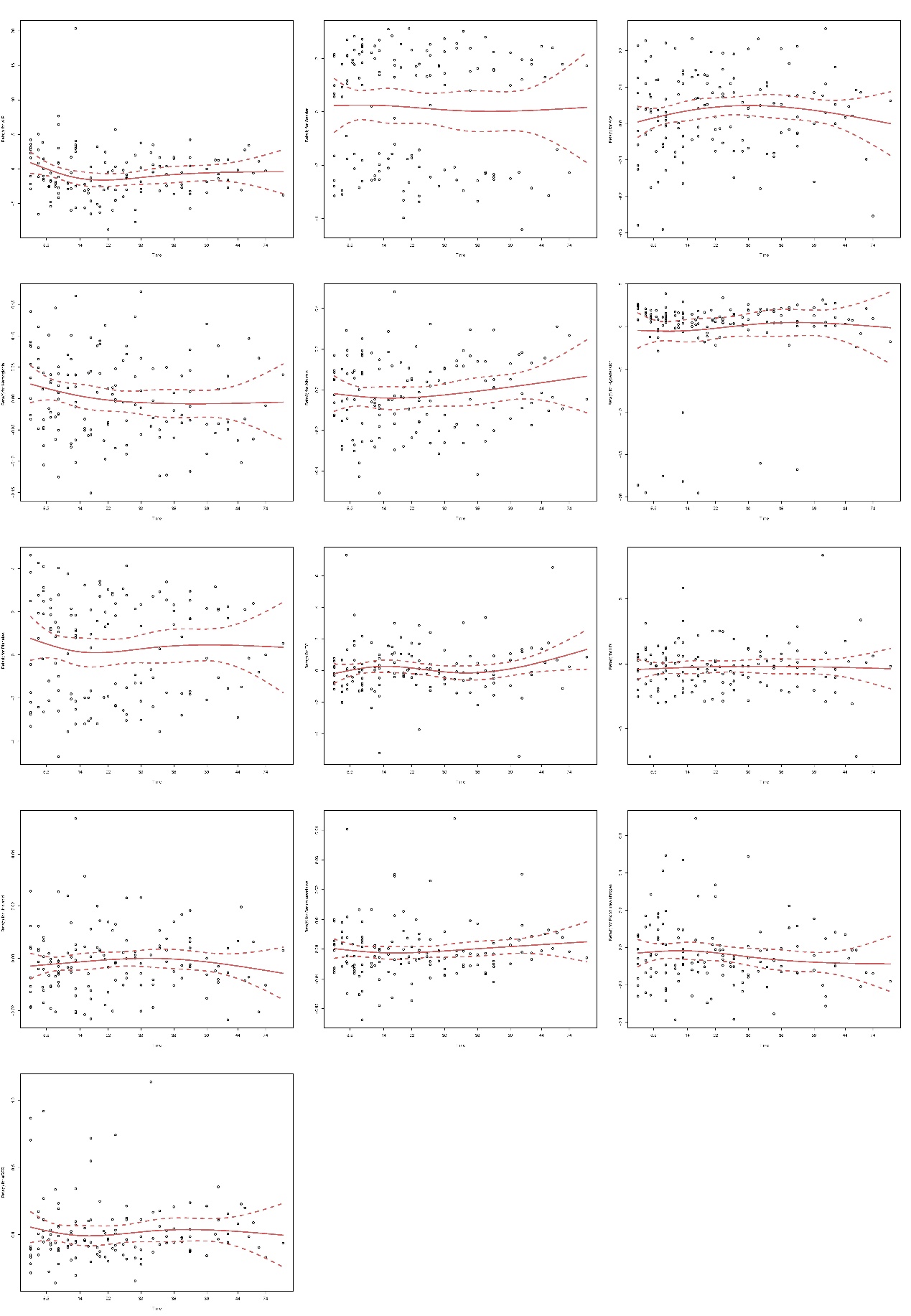


**Fig. S3 Visualization of Schoenfeld residuals testing**
